# OMI and Ground-Based In-Situ Tropospheric Nitrogen Dioxide Observations over Several Important European Cities during 2005–2014

**DOI:** 10.3390/ijerph14111415

**Published:** 2017-11-20

**Authors:** Spiru Paraschiv, Daniel-Eduard Constantin, Simona-Lizica Paraschiv, Mirela Voiculescu

**Affiliations:** 1 Department of Thermal Systems and Environmental Engineering, Faculty of Engineering, “Dunarea de Jos”, University of Galati, Str. Domneasca, Nr.111, 800008 Galati, Romania; sparaschiv@ugal.ro (S.P.); scraciun@ugal.ro (S.-L.P.); Voiculescu@ugal.ro (M.V.); 2 European Center of Excellence for the Environment, Faculty of Sciences and Environment, “Dunarea de Jos”, University of Galati, Str. Domneasca, Nr.111, 800008 Galati, Romania

**Keywords:** Ozone Monitoring Instrument, remote sensing, space observations, in-situ measurements, nitrogen dioxide, urban air quality

## Abstract

In this work we present the evolution of tropospheric nitrogen dioxide (NO_2_) content over several important European cities during 2005–2014 using space observations and ground-based in-situ measurements. The NO_2_ content was derived using the daily observations provided by the Ozone Monitoring Instrument (OMI), while the NO_2_ volume mixing ratio measurements were obtained from the European Environment Agency (EEA) air quality monitoring stations database. The European cities selected are: Athens (37.98° N, 23.72° E), Berlin (52.51° N, 13.41° E), Bucharest (44.43° N, 26.10° E), Madrid (40.38° N, 3.71° W), Lisbon (38.71° N, 9.13° W), Paris (48.85° N, 2.35° E), Rome (41.9° N, 12.50° E), and Rotterdam (51.91° N, 4.46° E). We show that OMI NO_2_ tropospheric column data can be used to assess the evolution of NO_2_ over important European cities. According to the statistical analysis, using the seasonal variation, we found good correlations (R > 0.50) between OMI and ground-based in-situ observations for all of the cities presented in this work. Highest correlation coefficients (R > 0.80) between ground-based monitoring stations and OMI observations were calculated for the cities of Berlin, Madrid, and Rome. Both types of observations, in-situ and remote sensing, show an NO_2_ negative trend for all of locations presented in this study.

## 1. Introduction

Nowadays, atmospheric pollution represents one of the most important threats to humans and life on Earth. Atmospheric pollution has been associated with a number of health problems, including heart disease, asthma, chronic obstructive pulmonary disease, lung cancer, etc. Urbanization associated with rapid industrial development has led to serious air pollution problems in many large cities across Europe with significant impact on public health and even on global climate. Air pollution in urban areas is one of the ten leading causes of death and illness worldwide for high income countries, with estimated one million deaths each year [1].

The transport, industry and production of thermal and electrical energy are main contributors to air pollution in Europe [2]. The emissions of major air pollutants have declined over the past decades which have led to a general improvement of air quality in Europe; however some sectors have not reduced their emissions sufficiently so as to meet the European Union (EU) air quality standards. Despite this fact, some domains recorded an increase in the emissions for certain pollutants [3]. As an example, the emissions of nitrogen oxides (NO_x_) from road transport have not decreased sufficiently to meet air quality standards in many urban areas; therefore the annual limit value for nitrogen dioxide (NO_2_) has been exceeded across Europe in 2014; approximately 94% of all of values above the annual limit value were measured at the traffic stations [4]. EU countries have taken various actions to reduce the emissions of air pollutants, but the results differ from one country to another. However, air quality standards are still outdated; therefore, further action is needed to reduce emissions of NO_2_ and other trace gases [4,5]. The NO_2_ concentrations in Europe are regulated by the Directive 2008/50/EC, designed to control specific concentrations of NO_2_ in the ambient air to which the European citizens are exposed. The directive specifies two limit values: 1-h limit value of 200 μg/m^3^ and a mean annual value of 40 μg/m^3^ [5].

NO_2_ is an important air quality indicator and a key component of urban air pollution. NO_2_ is a key atmospheric pollutant because of its health effects and also because it can absorb the visible solar radiation and contribute to impaired atmospheric visibility. If the concentration of NO_2_ were very high it could have a potential direct role in global climate change [6]. NO_2_ is a precursor of tropospheric ozone, solid particles and acid rain. Epidemiological studies have shown that the main adverse effects of NO_2_ on health include cardiopulmonary mortality, lung cancer and accentuation of asthma [6,7,8]. The lifetime of NO_2_ in the troposphere is short i.e., several hours, resulting in large spatial variability near sources [9]. The tropospheric NO_2_ has high spatial and temporal variability, mainly due to the change in the volume of local emissions, seasonal cycles and weather conditions [10,11,12].

In the last twenty years major advances have been made in detecting atmospheric pollution from space. Data provided by space-borne instruments have been used to monitor atmospheric pollutants, such as NO_2_. Space instruments, such us OMI (Ozone Monitoring Instruments) [13] or GOME-2 (Global Ozone Monitoring Experiment) [14] are used nowadays for the detection NO_2_ from space. Despite the fact that remote sensing from space is a very important tool in determining of NO_2_ from ground a synergetic view is necessary to assess air pollution by using complementary in-situ measurements or model simulations.

In this study, we present the evolution of NO_2_ content in the troposphere for several European cities using in-situ ground based information provided by EEA (European Environment Agency) and remote sensing observations derived from the OMI space instrument. In comparison to other studies [15,16,17,18,19,20,21,22], this study presents an updated image of NO_2_ of using ground-based measurements and remote sensing observation from space using existing databases for several cities, located in different areas, which be can representative for Europe. Also, we present correlations between ground and space observations for the selected cities.

The work is organized as follows: Data and Methodology are described in Section 2, while the Results and Discussions are presented in Section 3. Section 4 is dedicated to Conclusions.

## 2. Data and Methodology 

**Figure 1:**
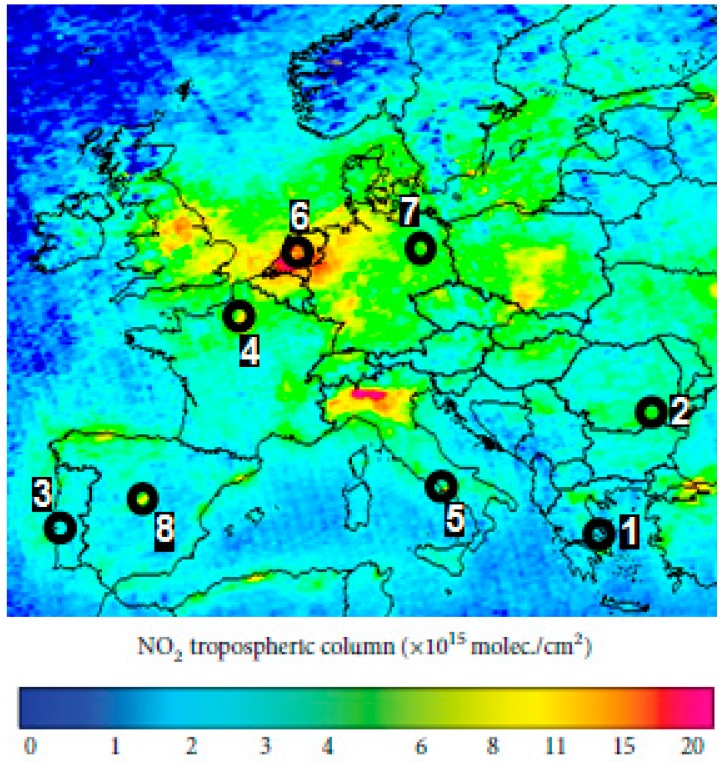
Spatial distribution of the selected cities using the 2014 Europe OMI NO_2_ map (Legend: 1—Athens, 2—Bucharest, 3—Lisbon, 4—Paris, 5—Rome, 6—Rotterdam, 7—Berlin, 8—Madrid).

Eight important urban agglomerations around the Europe were selected as focal points for this study (Figure 1). The selected cities are listed in Table 1 including information about geolocation and area.

The evaluation of the status and trends of NO_2_ over the selected cities were based on ground-based in-situ measurements and remote sensing space observations. The ground-based in-situ measurements used are data reported by the European countries to EEA, for the period 2005–2014. Daily ground-based in-situ observations provided by EEA are available until 2011, while the annual mean cover the entire period 2005–2014. The ground-based in-situ measurements are available via AirBase v.7—the European air quality database (EEA). The European NO_2_ monitoring network is usually based on instruments that use the chemiluminescence technique. A general view over the in-situ ground-based monitoring station presented in this study is introduced in Table 2. The ground-based in-situ NO_2_ measurements used in this work represent the annual average of the daily observations from the EEA database. Information about the monitoring stations used are presented in Table 2. Also, in this study, the average of observations recorded at traffic stations and urban stations will be used as “all stations”.

The remote sensing space observations used in this work are represented by OMI measurements. Data from the OMI instrument were used in this work, the space instrument provides more than 10 years of homogenous time series of NO_2_ observations. Despite the fact that OMI provides observations including 2017, starting in October 2004, to match the EEA database only the time interval 2005–2014 is presented in this work. The daily observations provided by EEA are available until 2011, while the annual mean is reported until 2014. The time interval 200–2014 will be used only for trend calculation and annual comparisons between OMI and ground-based observations, while the time interval 200–2011 will be used for seasonal time series comparisons.

OMI is a nadir UV-Vis spectrometer launched on-board of the Aura satellite on 15 July 2004. The OMI space equipment is one of the most advanced satellite measuring instruments, providing a daily dataset of tropospheric NO_2_ content with a spatial resolution of 13 × 24 km^2^ [13]. In order to analyze the evolution of tropospheric NO_2_ over the selected cities, the daily OMI NO_2_ overpass data available via TEMIS database (Tropospheric Emission Monitoring Internet Service) were used. The first product of OMI is a NO_2_ slant column density which must be converted to the tropospheric NO_2_ Vertical Column Density (VCD). The tropospheric VCDs are based on the DOMINO (Dutch OMI NO_2_) retrieval algorithm developed by KNMI (the Royal Netherlands Meteorological Institute), in collaboration with NASA (National Aeronautics and Space Administration). A full description of the retrieval algorithm can be found [23]. For this work we used the DOMINO version 2.0 (KNMI, Debildt, The Netherlands) daily tropospheric NO_2_ VCD overpass over the selected cities, available via www.temis.nl, filtered by a cloud fraction <30%. The number of OMI pixels selected for this study is direct proportional with the surface area of the urban area selected related to the pixel spatial resolution.

## 3. Results and Discussion

**Figure 2:**
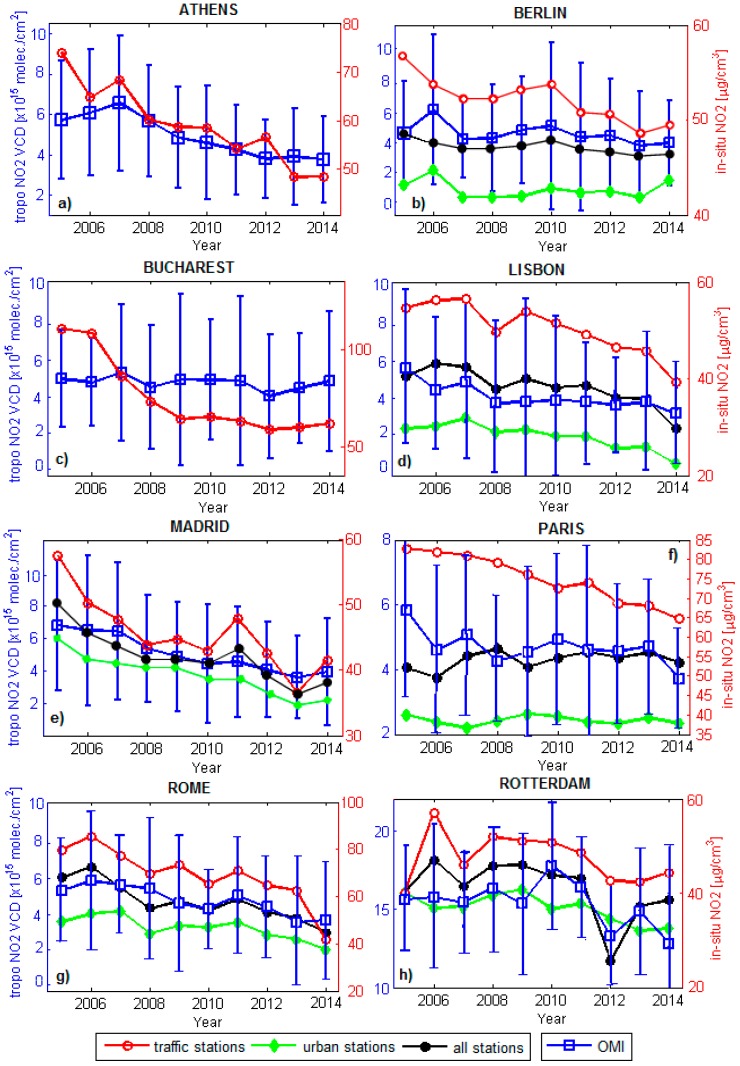
The time series of NO_2_ concentration from urban and traffic stations, including OMI NO_2_ observations for the selected cities: (**a**) Athens, (**b**) Berlin, (c) Bucharest, (**d**) Lisbon, (**e**) Madrid, (**f**) Paris, (**g**) Rome, (**h**) Rotterdam. “All stations” represents the average of traffic stations and urban stations.

In Europe, many cities, including those presented in this work, are experiencing severe air quality degradation due to urban development and increased industrialization; therefore, in order to implement effective strategies to reduce emissions, it is necessary to assess the spatio-temporal distribution of NO_2_ in these regions. The OMI space observations show a decreasing trend for the tropospheric NO_2_ content over all of the cities presented in this work (Figure 2). Analyzing the annual mean of tropospheric NO_2_ derived from OMI we observed that the highest tropospheric NO_2_ amount was recorded over the city of Rotterdam in 2010, ~18 ± 4.3 × 10^15^ molec./cm^2^, while the lowest tropospheric NO_2_ amount was recorded over the city of Lisbon (~3 ± 2.1 × 10^15^ molec./cm^2^) in 2014. The city of Rotterdam is located in the Benelux region where high tropospheric NO_2_ values are also reported by other studies [16,17,18,19,20]. Benelux region and North-West of Germany represent important NO_2_ hot-spot in Europe, which corresponds to highly industrialized regions.

Regarding the annual average concentrations of NO_2_ reported by the European local environmental agencies, the in-situ monitoring stations reported annual NO_2_ concentrations which generally are in the range of 20–110 μg/m^3^. The annual limit value of 40 μg/m^3^, imposed by the EU directives, was exceeded at all monitoring stations in this study, especially before 2010 (Figure 2). An interesting fact is that the global financial crisis 2007–2008 is visible for all of eight cities from any type of observations, either ground-based or space.

The first city analyzed in this study is Athens (Figure 2a), the capital and largest city of Greece. The air quality monitoring stations, available for this study, consist of four urban traffic stations. Figure 2a shows that the city of Athens is facing with a decreasing trend of tropospheric NO_2_ [24,25] starting in 2007, a fact which can be linked to the economic crisis, which seems to affect all of the NO_2_ sources of Athens, including road transport [26]. In 2014 the NO_2_ over Athens recorded by OMI shows a 43 ± 28% decrease compared to 2007, while the ground-based traffic stations show a ~30% decrease for the same period. Despite of this important NO_2_ decrease, the annual NO_2_ mean recorded at the traffic stations in Athens still exceeds the EU environmental directives.

The city of Berlin (Figure 2b) is the capital of Germany, it has a population of ~3.5 millions of inhabitants and a surface of 892 km^2^. The lowest NO_2_ content was observed by space and ground-based observation in 2007, year which corresponds to the great recession which starts in 2007. Using OMI observations we found a negative trend of ~1 ± 0.06% per year. Despite of fact that the in-situ traffic stations from Berlin show a decrease of ~15% in 2014, the annual mean recorded by the traffic stations is still over the annual limit of 40 μg/m^3^. During 2005–2014, the annual limit of 40 μg/m^3^ was exceeded for all Berlin’s monitoring stations presented in this study.

Bucharest city (Figure 2c) is the capital and largest city of Romania, located in the southeast of the country. It has a surface area of approximately 228 km^2^ and a population of ~2 million inhabitants. The air quality monitoring stations presented in this study consists of two urban traffic stations. The road traffic is the main source of NO_2_ pollution of Bucharest [27]. Figure 2c presents the evolution of NO_2_ over Bucharest during 2005–2014. We found that, during 2005–2007, the traffic monitoring stations recorded in Bucharest NO_2_ concentrations which exceeded even twice the NO_2_ annual limit value. However, in 2014 the urban traffic monitoring stations show an important decrease compared to 2007, ~45%. The significant reduction in traffic-related NO_2_ emissions can also be attributed to the national program to stimulate the renewal of the Romanian car fleet [27]. For the same period OMI shows only a ~10 ± 35% decrease. The discrepancy between OMI and the ground-based traffic stations is related to the fact that OMI takes in to consideration all of NO_2_ sources, also an OMI pixel (13 × 24 km^2^) can cover the full city of Bucharest not only the city center where usually the NO_2_ from traffic is focused.

Another city introduced in this study is Lisbon, the capital of Portugal. For this city we used eight monitoring stations available during 2005–2014. Compared to the other cities presented in this work, the city of Lisbon (Figure 2d), presents the lowest NO_2_ content as was observed by OMI. Using both types of observations, we found that the city of Lisbon has recorded negative NO_2_ trend of ~3 ± 0.05% per year. 

The city of Madrid (Figure 2e) is the main city of Spain, it has a population of ~3 millions of inhabitants and a surface of 604 km^2^. During 2005–2014 OMI recorded a ~36 ± 54% decrease for the tropospheric NO_2_ VCD, a similar percentage being calculated using the average of the NO_2_ monitoring traffic stations and urban stations. Until 2011, the annual average of the NO_2_ measurements recorded at the traffic and urban monitoring stations exceed the annual limit of 40 μg/m^3^.

In recent years, Paris has become known as a very polluted city in terms of emissions from motor vehicles [6]. The capital of France presents serious problems of pollution; regarding this issue the French government and the local authorities of Paris issued drastic measures to reduce the air pollution emitted by local traffic. The year 2005 presents the highest annual NO_2_ mean concentration recorded by the ground-based stations in Paris, ~85 μg/m^3^. The measures implemented by the French government and local authorities of Paris led in 2015 to an annual NO_2_ concentration of ~64 μg/m^3^, a visible fact also in the space observations which shows only 4.4 ± 2.8 × 10^15^ molec./cm^2^ for 2015 compared to 2005 when the annual mean observed by OMI was ~6.2 ± 4.9 × 10^15^ molec./cm^2^(Figure 2f).

The city of Rome is facing the same problems as Paris. The evolution of NO_2_ observed from space above Rome decreased by ~20 ± 56% during 2005–2014. A very pronounced reduction, of ~50%, is observed at the traffic monitoring stations (Figure 2g). The average of all of monitoring stations in Rome show a ~25% decrease of NO_2_. Despite of all efforts and measures implemented to reduce the air pollution in 2014, all of station from Rome exceeded the annual limit of 40 μg/m^3^, the case being similar to Paris and Athens.

The last city presented in this paper is Rotterdam, the second largest city in the Netherlands with an area of approximately 319 km^2^ and a population of ~650,000 inhabitants. The air quality monitoring system consists of two urban traffic stations and an urban station. The NO_2_ concentration measured at the ground stations, including the space observations, shows an ascending trend for the period 2005–2010 (~2 ± 0.05%/year), while after this period, until 2014, the trend is descending (~4±0.07%/year), (Figure 2h). 

**Figure 3:**
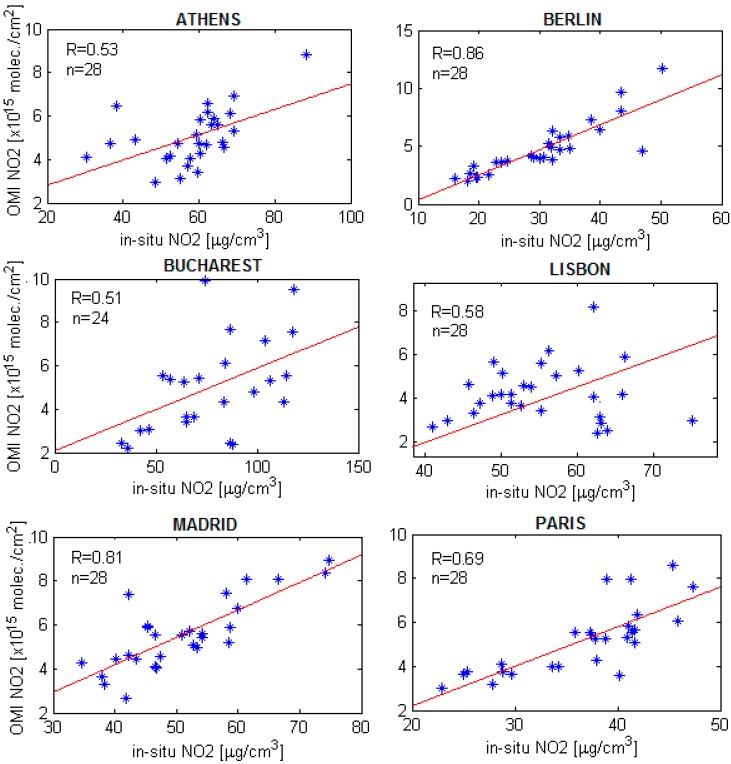

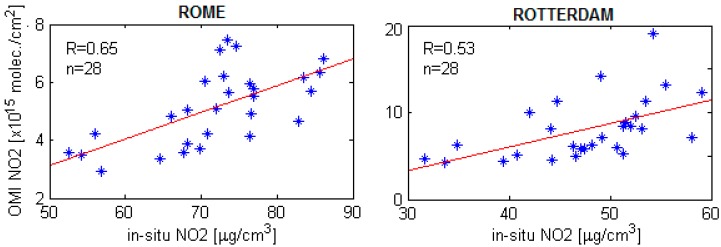
Correlations between the seasonal variation of tropospheric NO_2_ VCD observed by OMI and in-situ NO_2_ recorded at the monitoring traffic stations during 2005–2011.

Table 3 presents the correlation coefficients between NO_2_ OMI observations and the measurements performed by each type of ground-based in-situ station, including the average of all existing stations for each city. All of the correlation coefficients are higher than 0.50, with coefficients which range between 0.51 and 0.86. The in-situ ground-level NO_2_ seasonal cycle is well captured by OMI, this being an important factor for the high correlation coefficients obtained. The high correlation coefficients between OMI observations and traffic station measurements can reinforce the idea that road traffic is an important contributor on the total budget of urban NO_2_. Correlations between tropospheric NO_2_ VCD observed by OMI and the in-situ NO_2_ recorded at the monitoring traffic stations during 2005–2011 are presented in Figure 3.

## 4. Conclusions

In Europe, cities in France and Italy are experiencing severe air quality degradation due to urban development and increased industrialization; therefore, in order to implement effective strategies to reduce emissions, it is necessary to assess the spatio-temporal distribution of NO_2_ and other air pollutants. In order to achieve an improvement in air quality and to implement appropriate measures it is absolutely necessary to understand local manifestations and evolution of various pollutants in the long run using ground-based or space observations.

In this paper we presented the evolution of tropospheric NO_2_ amount over several important European cities during 2005–2014 using space observations and ground-based in-situ measurements. Using NO_2_ from ground measurements and space observations we found an NO_2_ descending trend of 2–5% per year for the selected cities. Despite of this descending trend for many cities the annual mean limit value of 40 μg/m^3^ is still exceeded. Our analysis showed that NO_2_ emissions from traffic are a key factor contributing to the exceedance of the limit value required by EU directives.

The annual average concentrations of NO_2_ in the urban areas of the cities analyzed are generally in the range of 20–110 μg/m^3^. We found very high NO_2_ annual concentrations at the traffic stations of Paris and Rome, which make the city of Paris and Rome some of the most polluted cities in Europe. Using OMI observations, we found the maximum annual mean value of tropospheric NO_2_ over the city of Rotterdam, ~18 ± 4.3 × 10^15^ molec./cm^2^, while the lowest tropospheric NO_2_ amount was recorded over the city of Lisbon (~3 ± 2.1 × 10^15^ molec./cm^2^) in 2014. Also, we presented correlations between OMI observations and ground-based in-situ measurements recorded at urban and traffic station, all of the correlations coefficients calculated are higher than 0.50. 

## Figures and Tables

**Table 1:** Information about the selected cities.

City	Country	Location	Area
Athens	Greece	37.98° N, 23.72° E	412 km^2^
Berlin	Germany	52.51° N, 13.41° E	892 km^2^
Bucharest	Romania	44.43° N, 26.10° E	228 km^2^
Lisbon	Portugal	38.71° N, 9.13° W	958 km^2^
Madrid	Spain	40.38° N, 3.71° W	604 km^2^
Paris	France	48.85° N, 2.35° E	2845 km^2^
Rome	Italy	41.9° N, 12.50° E	1285 km^2^
Rotterdam	The Netherlands	51.91° N, 4.46° E	326 km^2^

**Table 2:** Information about the type and number of available monitoring stations used in this study for the period 2005–2014.

Location	Athens	Bucharest	Berlin	Lisbon	Madrid	Paris	Rome	Rotterdam
Number of available traffic stations	4	2	4	4	6	7	5	2
Number of available urban stations	0	0	4	4	5	9	7	0

**Table 3:** Correlation coefficients between OMI and ground-based in-situ measurements using the seasonal variation of NO_2_.

City	Correlation Coefficient (R) for Each Type of Station		
	T *	U *	A *
Athens	0.53	-	-
Berlin	0.86	0.84	0.87
Bucharest	0.51	-	-
Lisbon	0.58	0.68	0.62
Madrid	0.81	0.75	0.86
Paris	0.69	0.74	0.79
Rome	0.65	0.86	0.81
Rotterdam	0.53	-	-

* where T = traffic, U = urban, A = the average of traffic and urban stations.

## References

[1] World Health Organization (2009). Global Health Risks: Mortality and Burden of Disease Attributable to Selected Major Risks.

[2] Martin B., Martin L. (2012). Association between unemployment, income, education level, population size and air pollution in Czech cities: Evidence for environmental inequality? A pilot national scale analysis. Health Place.

[3] Susannah G., John S., Andrew K., Melanie H., John N., John A., Sally C. (2009). Recent trends and projections of primary NO_2_ emissions in Europe. Atmos. Environ..

[4] European Environment Agency (2015). Air Quality in Europe—2015 Report.

[5] European Environment Agency (2016). Air Quality in Europe—2016 Report.

[6] World Health Organization (2006). WHO Air Quality Guidelines for Particulate Matter, Ozone, Nitrogen Dioxide and Sulfur Dioxide.

[7] Song X., Liu Y., Hu Y., Zhao X., Tian J., Ding G., Wang S. (2016). Short-Term Exposure to Air Pollution and Cardiac Arrhythmia: A Meta-Analysis and Systematic Review. Int. J. Environ. Res. Public Health.

[8] Latza U., Gerdes S., Baur X. (2009). Effects of nitrogen dioxide on human health: Systematic review of experimental and epidemiological studies conducted between 2002 and 2006. Int. J. Hyg. Environ. Health.

[9] Berezin E.V., Konovalov I.B., Romanova Y.Y. (2016). Inverse Modeling of Nitrogen Oxides Emissions from the 2010 Russian Wildfires by Using Satellite Measurements of Nitrogen Dioxide. Atmosphere.

[10] Zhou Y., Dominik B., Christoph H., Stephan H., Johannes S. (2012). Changes in OMI tropospheric NO_2_ columns over Europe from 2004 to 2009, and the influence of meteorological variability. Atmos. Environ..

[11] Qi Y. (2015). Spatio-temporal distributions of tropospheric NO_2_ over oases in Taklimakan Desert, China. Chin. Geogr. Sci..

[12] Zhang L., Lee C.S., Zhang R., Chen L. (2017). Spatial and temporal evaluation of long term trend (2005–2014) of OMI retrieved NO_2_ and SO_2_ concentrations in Henan Province, China. Atmos. Environ..

[13] Levelt P., van den Oord G., Dobber M., Malkki A., Visser H., de Vries J., Stammes P., Lundell J., Saari H. (2006). The ozone monitoring instrument. IEEE Trans. Geosci. Remote.

[14] Munro R., Eisinger M., Anderson C., Callies J., Corpaccioli E., Lang R., Lefebvre A., Livschitz Y., Albinana A.P. GOME-2 on MetOp. Proceedings of the 2006 EUMETSAT Meteorological Satellite Conference.

[15] Schaap M., Kranenburg R., Curier L., Jozwicka M., Dammers E., Timmermans R. (2013). Assessing the Sensitivity of the OMI-NO_2_ Product to Emission Changes across Europe. Remote Sens..

[16] Szymankiewicz K., Kaminski J.W., Struzewska J. (2014). Interannual variability of tropospheric NO_2_ column over Central Europe—Observations from SCIAMACHY and GEM-AQ model simulations. Acta Geophys..

[17] Miranda A., Silveira C., Ferreira J., Monteiro A., Lopes D., Relvas H., Borrego C., Roebeling P. (2015). Current air quality plans in Europe designed to support air quality management policies. Atmos. Pollut. Res..

[18] Constantin D.E., Voiculescu M., Georgescu L. (2013). Satellite Observations of NO_2_ Trend over Romania. Sci. World J..

[19] Zyrichidou I., Koukouli M.E., Balis D.S., Katragkou E., Melas D., Poupkou A., Kioutsioukis I., van der A. R., Boersma F.K., van Roozendael M. (2009). Satellite observations and model simulations of tropospheric NO_2_ columns over South-Eastern Europe. Atmos. Chem. Phys..

[20] Schneider P., Lahoz W.A., van der A R. (2015). Recent satellite-based trends of tropospheric nitrogen dioxide over large urban agglomerations worldwide. Atmos. Chem. Phys..

[21] Castellanos P., Boersma K.F. (2012). Reductions in nitrogen oxides over Europe driven by environmental policy and economic recession. Sci. Rep..

[22] Guerreiro C.B., Foltescu V., de Leeuw F. (2014). Air quality status and trends in Europe. Atmos. Environ..

[23] Boersma K.F., Eskes H.J., Dirksen R.J., van der A R.J.J., Veefkind P., Stammes P., Huijnen V., Kleipool Q.L., Sneep M., Claas J. (2011). An improved retrieval of tropospheric NO_2_ columns from the Ozone Monitoring Instrument. Atmos. Meas. Tech..

[24] Varotsos C., Christodoulakis J., Tzanis C., Cracknell A.P. (2014). Signature of tropospheric ozone and nitrogen dioxide from space: A case study for Athens, Greece. Atmos. Environ..

[25] Tzanis C., Varotsos C., Ferm M., Christodoulakis J., Assimakopoulos M.N., Efthymiou C. (2009). Nitric acid and particulate matter measurements at Athens, Greece, in connection with corrosion studies. Atmos. Chem. Phys..

[26] Sidiropoulou T., Ioannis P. (2015). Austerity and Fuel Consumption in Greece: An Empirical Investigation. Int. J. Econ. Bus. Adm..

[27] Constantin D.-E., Voiculescu M., Georgescu L., Trif C., Karakolios E., Mamoukaris A., Xipolitos K. (2012). Imprint of Road Vehicle Dynamics on Atmospheric Pollution. Case Study: Bucharest City 2007–2010. J. Environ. Prot. Ecol..

